# Products for Sportspeople Containing Constituents Derived from the Common Bean *Phaseolus vulgaris* L. (Fabaceae)—A Narrative Literature Review

**DOI:** 10.3390/sports11110211

**Published:** 2023-10-31

**Authors:** Kinga Kostrakiewicz-Gierałt

**Affiliations:** Department of Tourism Geography and Ecology, Institute of Tourism, Faculty of Tourism and Recreation, University of Physical Education in Kraków, Jana Pawła II 78, 31-571 Cracow, Poland; kinga.kostrakiewicz@awf.krakow.pl

**Keywords:** athletes, endurance, health, nutrition, patents

## Abstract

The third-largest land plant family, Fabaceae (Papilionaceae), includes trees, shrubs, and perennial or annual herbaceous plants containing both numerous beneficial constituents (e.g., proteins, carbohydrates, dietary fibre) and antinutrients (e.g., saponins, tannins, phytic acid, gossypol, lectins). The consumption of leguminous plants allows sports people to complete their requirements for nourishment but, on the other hand, it contributes to digestive system ailments. Therefore, the aim of the presented study was to review the experimental articles and patents referring to the application of common (kidney) bean (*Phaseolus vulgaris* L.)-based nutritional products for athletes. The survey of the literature was carried out according to PRISMA statements by browsing Scopus, PubMed and ISI Web of Science databases, as well as Google Scholar, Google Patents and Espacenet Patent Search engines using factorial combinations of the following keywords: (‘common bean’ or ‘kidney bean’ or ‘*Phaseolus vulgaris*’) and (‘athlete’ or ‘sport’) and (‘food’ or ‘nutrition’ or ‘diet’). Altogether, 84 patents issued in the years 1995–2023 were noted. The majority of patents were developed by research teams consisting of at least four authors representing scientists affiliated in the United States of America and China. The patents refer to the production of food ingredients, nutritional products, and compositions: (i) for relieving fatigue, enhancing endurance, and increasing muscle mass and strength, (ii) for maintaining physical and mental health, and (iii) for controlling body weight. Moreover, the analysis of 19 original articles indicated the substantial acceptability of meals containing the common bean. To summarize, the performed investigations demonstrate the considerable use of *Phaseolus vulgaris* in sport nutrition and the growing acceptance of this trend.

## 1. Introduction

The botanical family of dicotyledonous plants, Fabaceae (Papilionaceae), includes trees, shrubs, and perennial or annual herbaceous plants, which are easily recognised by their papilionaceous flowers, fruits, and compound, stipulate leaves. The family is the third-largest land plant family in terms of number of species, behind only the Orchidaceae and Asteraceae, with more than 720 genera and nearly 20,000 known species [1]. The leguminous plants develop a symbiotic relationship with nitrogen-fixing bacteria which invade the root hairs of host plants, multiply, and stimulate the formation of root nodules. Within the nodules, microorganisms convert free nitrogen to ammonia, which the host plant utilises for its development. The nitrogen fixation is possible thanks to sugars created in photosynthesis and provided by the plant. Nodulated legumes are found in all environments except open seas and are arguably more significant at high than low latitudes [2]. A detailed biogeography of nodulated legumes and their nitrogen-fixing symbionts is presented by Sprent et al. [3].

Due to the additional nitrogen that legumes receive through the process of atmospheric nitrogen fixation, they have a comparatively higher amino acid content than other species [4]. Moreover, they contain carbohydrates, dietary fibre, fat, and minerals, such as calcium, copper, iron, magnesium, phosphorus, potassium, and zinc [5,6,7]. They provide tremendous opportunities and challenges for use in processed foods such as bakery products, bread, pasta, soaked and dried foods, snack foods, soups, cereal bar filings, tortillas, meat, etc. [8]. According to numerous authors, the consumption of legumes is suitable for sportspeople who follow different diets and present diverse eating habits, allowing them to complete their requirements for nourishment [9,10,11,12,13]. Tukhtarov [9] stated that the consumption of legumes enhances the total biological value of the average daily diets of professional athletes in Uzbekistan. Rogerson [10] argued that beans are a rich source of proteins, carbohydrates, and iron, which are very important for sportspeople who follow a vegan diet. D’Angelo and Cusano [11] highlighted the value of legume consumption by sportspeople following the Mediterranean diet. Additionally, Terenzio et al. [12] pointed out that the consumption of legumes improves the sport performance of Italian athletes. Shevkani et al. [13] documented the beneficial role of proteins from leguminous plants in the qualitative improvement of gluten-free foods suitable for sportspeople with gluten-related disorders. On the other hand, despite their benefits, legumes contain antinutrients such as saponins, tannins, phytic acid, gossypol, lectins, protease inhibitors, amylase inhibitors, and goitrogens. Anti-nutritional factors combine with nutrients and are a major concern because of the resulting reduced nutrient bioavailability [14]. Moreover, the substantial consumption of legume plants may contribute to digestive system ailments such as the feeling of fullness, discomfort in the stomach, bloating, and diarrhoea. The avoidance of legume consumption by athletes with gastrointestinal disorders has been advised [15] and confirmed [16] in studies.

Considering the aforementioned discrepancy, it would seem to be very interesting to conduct a review of the use of particular species representing the Fabaceae family in the nourishment of sportspeople. So far, such investigations have been conducted in the case of the soybean, *Glycine max* (L.) Merr. [17].

*Phaseolus vulgaris* is another species representing the Fabaceae family characterized by the presence of both beneficial substances, as well as the antinutrients and allergens. The beneficial substances contribute to numerous health benefits, e.g., diminished heart and renal disease risks, increased satiation, and cancer prevention. Moreover, the intake of meals comprising seeds of *Phaseolus vulgaris* by sportspeople contributes to meeting energy needs and to rapid recovery after injury. On the other hand, undesirable components such as antinutrients may diminish the bioavailability of trace elements, as well as limit protein and carbohydrate utilization. In light of this dichotomy, a review of the literature regarding the use of alimentary products containing *Phaseolus vulgaris* by sportspeople is strongly desirable. At the same time, it should be added that a review of patents for beverages enriched with plant proteins derived among others from *Phaseolus vulgaris* and intended for use by athletes was presented by Arbach et al. [18]. Nevertheless, the current state of knowledge on the use of the common bean in sport nourishment is insufficient; therefore, the present investigations were undertaken. The main objectives were to learn:

(i) Which constituents of *Phaseolus vulgaris* are used in food products dedicated to sportspeople?
(ii) What are the effects of the activity of *Phaseolus vulgaris* constituents in food products?
(iii) What is the frequency and time of intake of food products by sportspeople?

## 2. Materials and Methods

### 2.1. Study Species

The common bean *Phaseolus vulgaris* L. [19] can grow as an annual in temperate climates and as an annual or short-lived perennial in tropical climates. The species is highly variable, from erect bushes measuring 20–60 cm in height to running vines reaching 2–3 m in length. All varieties bear alternate green or purple leaves, which are divided into three oval, smooth-edged leaflets, each 6–15 cm long and 3–11 cm wide. The white, pink, or purple flowers are about 1 cm long, and they give way to pods 8–20 cm long and 1–1.5 cm wide. These may be green, yellow, black, or purple, each containing 4–6 oblong-ellipsoid beans. The beans are smooth, plump, kidney-shaped, up to 1.5 cm long, ranging widely in colour and are often mottled in two or more colours.

The domestication of *Phaseolus vulgaris* occurred independently in the Mesoamerican and Andean areas, which gave rise to two highly differentiated gene pools. The Mesoamerican common bean probably arrived in Europe through Spain and Portugal in 1506, and the Andean in the same way in 1528, after the exploration of Peru by Pizarro. The genetic structure of European populations is presented by Angioi et al. [20]. Due to extensive plant-breeding efforts, *Phaseolus vulgaris* L. comprises numerous cultivars (e.g., navy bean, pinto bean, red kidney bean, black kidney bean, white kidney bean) with a wide range of morphological and agronomic characteristics, including differences in seed size and colour as well as growth habit [21].

*Phaseolus vulgaris* L. is a source of several constituents beneficial for human health such as protein, carbohydrates, essential fatty acids, vitamins, minerals, and fibres. Besides these nutrients, the common bean is also rich in bioactive compounds, such as polyphenols, mainly flavonoids. The role of *Phaseolus vulgaris* L. in the prevention of diabetes [22,23,24,25,26], metabolic syndrome [23,25], obesity [26,27,28], many types of cancer [22,23,24,25,26], and cardiovascular diseases [22,23,29,30] has been extensively reported. According to many authors, e.g., [31,32,33,34,35,36], the presence of the common bean in the diet contributes to meeting energy, macronutrient, and micronutrient needs by sportspeople to keep them ready to play and/or return to play after injury. At the same time, the occurrence of allergens and antinutrients, such as lectins, phytic acid, and condensed tannins in the seeds of the common bean, has been pointed out e.g., [37,38,39].

### 2.2. Collection Procedure

#### 2.2.1. Literature Search

The author searched for peer-reviewed original full-text articles and patents regarding the application of a kidney bean diet for sportspeople using Scopus, PubMed, and ISI Web of Science-indexed publications. These search engines were selected as they provide a comprehensive all-encompassing database for various interdisciplinary domains. Moreover, publications were searched by browsing Google Scholar. Patents were searched by browsing the Google Patents and Espacenet Patent Search engines, because they gather the largest number of open access patents. The review focused on literature records published up to 2023. The author used factorial combinations of the following keywords in the searches: (‘common bean’ or ‘kidney bean’ or ‘*Phaseolus vulgaris*’) and (‘athlete’ or ‘sport’) and (‘food’ or ‘nutrition’ or ‘diet’). The selection terms were examined from the title, abstract, and keywords of the articles. The literature search was conducted from 1 May to 1 July 2023. The results included 87 hits from the ISI Web of Science, 97 hits from Scopus, 1042 from PubMed, 1061 from Google Scholar, 1157 from Google Patents, and 4687 from Espacenet. After the manual removal of grey literature (blog posts, letters, manuals, guides, bulletins, newsletters, editorials, commentaries, theses, dissertations, reports, conference proceedings, and meeting notes) from the lists of searches, the patents and peer-reviewed articles were selected. 

#### 2.2.2. Study Eligibility and Selection

Following the removal of duplicates (publications indexed in at least two databases), the abstracts of patents and articles were screened for relevance and eligibility. 

The inclusion criteria for patents were: (i) they were useful for sport practitioners and (ii) the abstract was written in English. The inclusion criteria for articles were as follows: (i) the studies were relevant to the main subject of the presented review, (ii) the participants were people, (iii) there were no limits regarding the age, weight, sex, nationality, or number of participants, (iv) there were no limits in geographical location or time period of investigations, (v) the publications presented original studies, (vi) there were no limits in dose, duration, delivery method of intervention, (vii) there were no limits regarding outcomes, and (viii) the abstract was written in English. The exclusion criteria for patents were: (i) the patents were not useful for sport practitioners, (ii) the abstract was not written in English. The exclusion criteria for articles were as follows: (i) the studies were irrelevant to the main subject, (ii) the investigations were conducted on non-human species, (iii) the publications were repetitive (different parts of a single study were presented in two or more papers or studies based on a population that was part of an earlier publication), (iv) the investigations were meta-analyses, or systematic reviews, and (v) there was no abstract in English.

Finally, a full-text screening was performed. The inclusion criteria for patents were: (i) they were useful for sport practitioners, (ii) there were no limits regarding the applied methods and outcomes, and (iii) the full text was written in English. The inclusion criteria for articles were as follows: (i) they were observational, descriptive studies (case report/case series), or (ii) they were observational, analytical studies (case–control studies, cross-sectional studies, cohort studies), or (iii) they were experimental studies (randomised controlled trials), (iv) there were no limits in dose, duration, delivery method of intervention, (v) there were no limits regarding outcomes, and (vi) the full text was written in English. The exclusion criteria for patents were: (i) the patents were irrelevant to the main subject, (ii) the patents were dedicated to non-human species, and (iii) the full text was not written in English. The exclusion criteria for articles were as follows: (i) in the Results section meta-analyses, overviews or systematic reviews were presented, (ii) there was no available full text, and (iii) the full text was not written in English.

#### 2.2.3. Data Extraction and Synthesis

**Figure 1:**
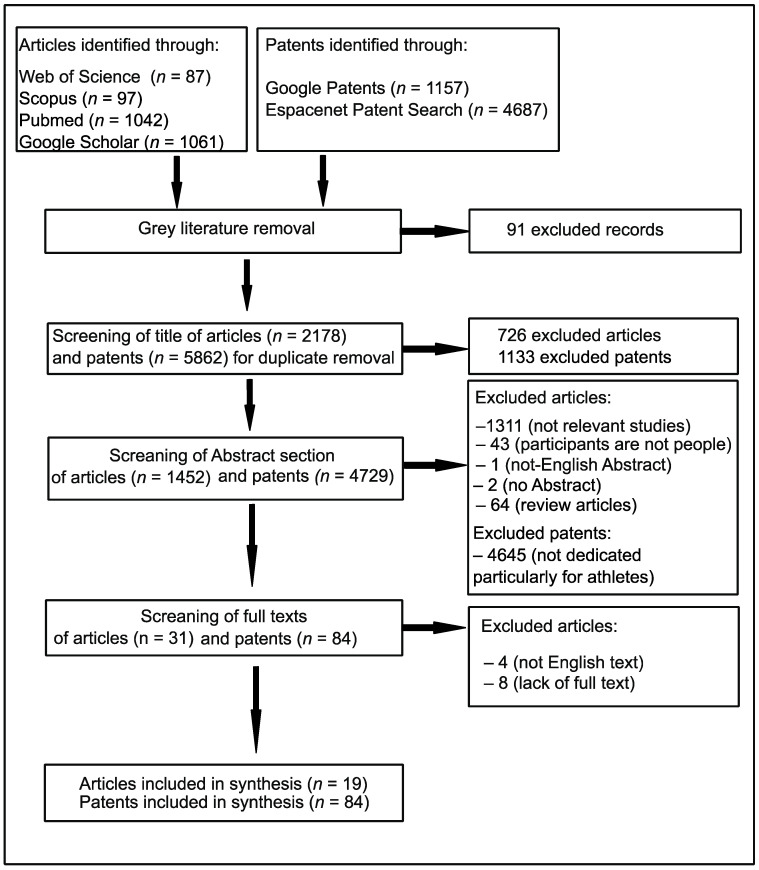
The procedure for the literature search according to Moher et al. [40].

To assess the quality of the included studies, all patents and articles were subjected to critical double screening. The following data were extracted: authors, affiliation of first author, year of publication, title, and characteristic of invention (in the case of patents), as well as authors, title and year of publication, and the age, gender, and nationality of participants and study findings (in the case of articles). A final total of 84 patents and 19 original articles were selected to be reviewed. A chart detailing the search results is presented in Figure 1. Results are reported in a narrative synthesis, using a textual approach to analyse the relationships within and between studies.

The statistical significance of differences in the number of patents in the study periods 1995–2001, 2002–2008, 2009–2015 and 2016–2023 was checked using the non-parametric Kruskal–Wallis H test.

## 3. Results

### 3.1. An Overview of Patents

**Figure 2:**
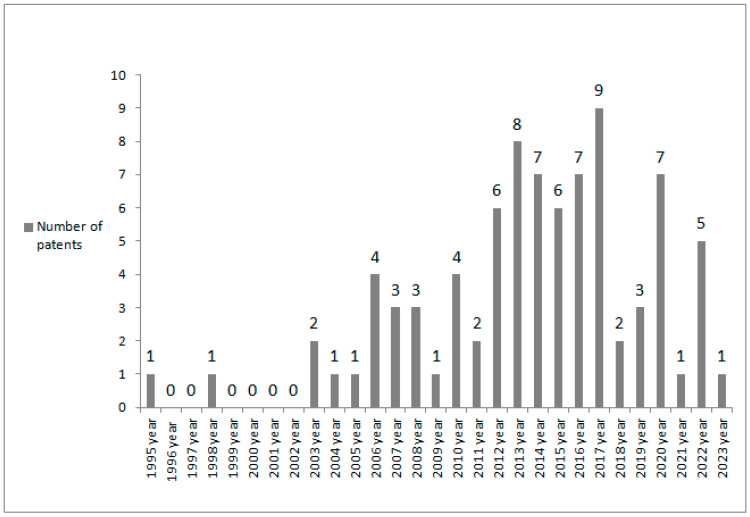
The number of patents containing constituents deriving from *Phaseolus vulgaris* L. suitable for sportspeople issued in the years 1995–2023.

Altogether, 84 patents issued in the years 1995–2023 were noted. The number of patents increased systematically in consecutive years up to the year 2017, when the greatest number of inventions was patented. During subsequent years, a slight decrease in the number of patents was observed (Figure 2). Regarding the number of patents issued in consecutive periods, it can be stated that significant differences were noted between the period 1995–2001 (when the lowest number of patents was issued) and the period 2010–2016 (when the greatest number of patents was observed) as well as the period 2017–2023 (when a considerable number of patents was noted) (Table 1).

**Figure 3:**
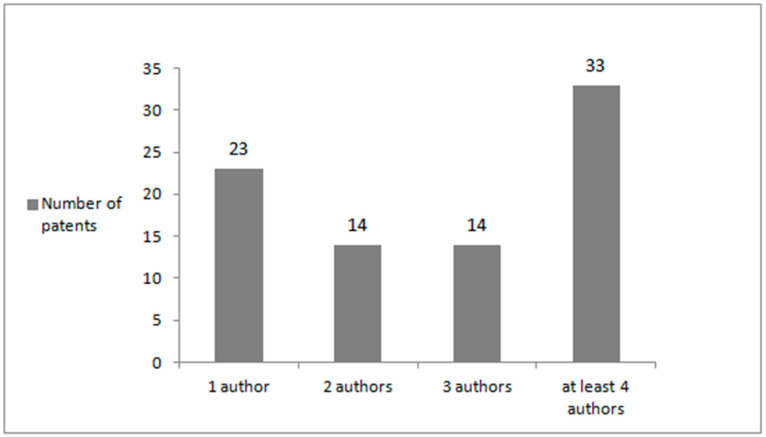
The number of patents containing constituents derived from *Phaseolus vulgaris* L. suitable for sportspeople obtained in the years 1995–2023 by one author or research teams consisting of two, three or at least four authors.

**Figure 4:**
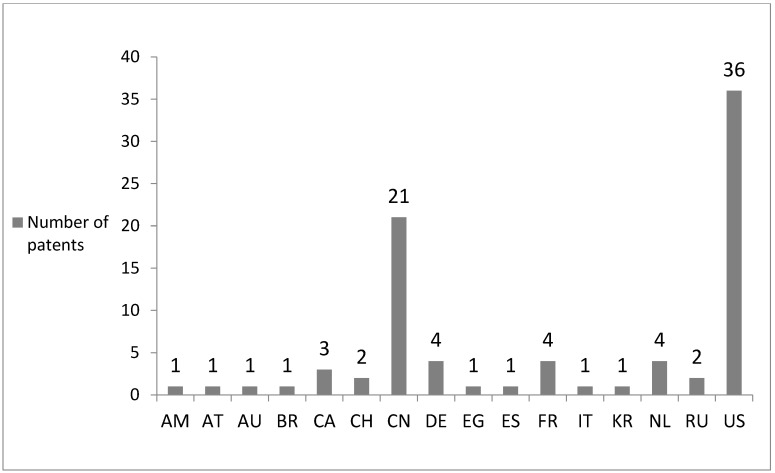
The number of patents containing constituents derived from *Phaseolus vulgaris* L. suitable for sportspeople obtained by authors affiliated in Armenia (AM), Austria (AT), Australia (AU), Belgium (BE), Brazil (BR), Canada (CA), Switzerland (CH), China (CN), Germany (DE), Egypt (EG), Spain (ES), France (FR), Italy (IT), Japan (JP), South Korea (KR), The Netherlands (NL), the Russian Federation (RU), the United States of America (US).

**Figure 5:**
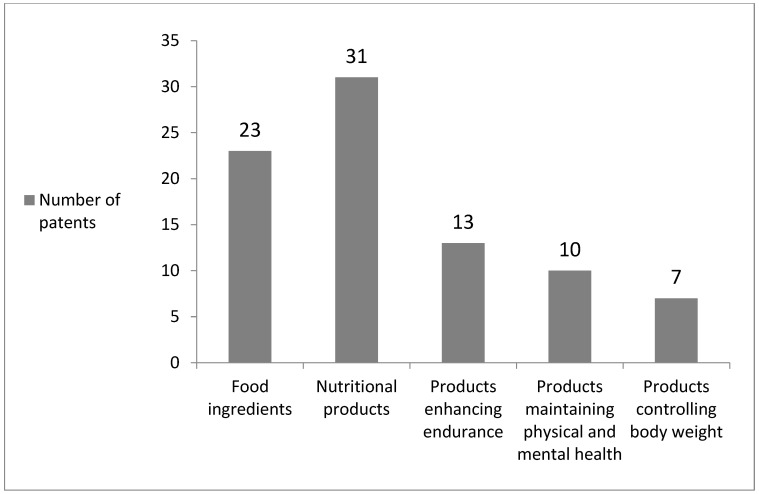
The number of patents containing constituents derived from *Phaseolus vulgaris* L. suitable for sportspeople as food ingredients, nutritional products, as well as nutritive compositions enhancing endurance, maintaining physical and mental health, and controlling body weight.

The majority of patents (39.29%) were obtained by research teams consisting of at least four authors, 27.38% were prepared by one author, while 16.67% were created in cooperation between two or three authors (Figure 3). Altogether, the authors were affiliated with 16 countries. The majority of patents (42.86%) were obtained by scientists representing the United States of America and researchers from China (25.00%). Moreover, 4.76% of patents were obtained by authors from Germany, France, and The Netherlands, whereas 3.57% patents were obtained by scientists from Canada, and 2.38% patents were obtained by researchers from Switzerland and the Russian Federation. The share of authors representing other countries reached 1.19% (Figure 4). Summing up, 27.38% of patents refer to the production of food ingredients. The remaining patents are devoted to nutritional products (36.90%), as well as nutritive compositions: (i) for relieving fatigue, for enhancing endurance as well as muscle mass and strength (15.48%), (ii) for maintaining physical and mental health (11.90%), and (iii) for controlling body weight (8.33%) (Figure 5).

### 3.2. The Characteristics of Patent Activity

Numerous patents refer to the use of constituents derived from *Phaseolus vulgaris* as food ingredients (Table 2). The majority of patents present methods of isolation of the amino acids [41], polypeptides [42], and proteins [43,44,45,46,47,48,49], as well as protein compositions [50] present in *Phaseolus vulgaris*. Boursier and Passe [51] patented a method for production of a granulated powder containing at least one protein of vegetable origin and at least one starch hydrolysate, which can be used in nutritional products suitable for, among others, athletes. Van der Hijden et al. [52] patented a method for the preparation of starch that has controlled energy release properties derived from leguminous species. Moreover, Slimak [53] patented an application of legumes such as *Phaseolus vulgaris* in flour production and its use in creating valuable edible products.

Kannar et al. [54] patented a process for manufacturing sugar products with desired levels of specific phytochemicals derived, among others, from pulses such as *Phaseolus vulgaris*. Several researchers [55,56,57] developed recipes for sweetener compositions comprised of one or more steviol glycosides and catechins or isoflavons derived from the kidney bean. Other authors [58,59,60,61] developed compositions modifying taste containing proanthocyanidins and procyanidins derived from legumes, among others, from *Phaseolus vulgaris*. Eidenberger [62] developed methods to prepare a stable anthocyanin composition which does not undergo degradation. Additionally, Hossen et al. [63] described methods of processing raw leguminous materials such as fruits or seeds to reduce non-volatile flavour components and, in particular, bound saponin compounds.

Many authors have developed recipes for a variety of products from nutritional supplements of small forms (e.g., bars and others) through sweet or savoury fillings and instant powders to wholesome meals (Table 3). A considerable number of nutritive compositions for sportspeople, such as bars [64], biscuits [65,66], cakes [67], chips [68], and frozen sweets [69], have been presented. Other scientists have patented nutritive sport drinks [70,71,72,73,74,75,76,77,78,79]. Smith, et al. [80] patented a functional food paste, which can be used as a nougat-like filling for bars, cookies, and cupcakes, as well as a savoury filling for baked products (e.g., crackers, pretzels, or bread). Protein-rich, multi-element powders containing, among others, cereals were invented by Hongtao [81], Li [82], and Lei et al. [83], whereas quinoa powder invented by Tao and Ting [84] is suitable for meal replacement. Other authors have developed recipes for nutritional compositions administered in various forms containing at least one dairy protein and at least one vegetable protein [85], including branched chain fatty acids, probiotics, nucleotides and amino acids [86], as well as plant-based protein mixtures [87]. Hangjian [88] prepared a method for the preparation of nutritious food with an obvious meal replacement effect, rich nutrition, good taste and easy portability composed of konjac refined powder, soybean protein, and/or ovalbumin, vitamins, trace elements and auxiliary materials such as vegetable powder (e.g., common bean powder). Xu [89] invented a method for preparing a nutritional food comprising corn flour, Astragalus root powder, small red bean powder, flour, propolis, honey, aspartame, and vinegar. Savant et al. [90] patented a nutritional puree composition comprising fruits and vegetables, inter alia the common bean. Nardelli [91] invented a nutritional dietary supplement formulated on the basis of moist cassava or manioc starch, which might be used by athletes as a partial meal replacement.

Numerous patents containing constituents derived from *Phaseolus vulgaris* are intended to relieve fatigue and enhance endurance, as well as increase muscle mass and strength (Table 4). Several authors have patented anti-fatigue products such as beverages comprising water, fructose syrup, edible salt, edible spices, and food additives [92] and functional pastes containing inter alia granulated sugar, soybean oil, and xanthan gum [93]. Hageman et al. [94] and De Wilde et al. [95] patented the compositions relieving perceived fatigue after exercise and methods for their application. Other authors have patented beverages relieving fatigue and improving endurance [96,97,98]. Other researchers [99,100] have developed recipes for products enhancing athletic performance and motor abilities. Xu [101] patented a beverage improving exercise intensity and endurance, and helping muscle recovery. Additionally, Bailey et al. [102] presented methods for enhancing muscle protein synthesis following physical exertion. Veen and Budemann [103] patented a food composition containing amino acids, which increases muscle mass and power and facilitates their faster recovery. In turn, Longo et al. [104] patented a diet enhancing muscle mass, which is usually desirable in sport training, competitive sports, and bodybuilding.

Many authors have obtained patents focusing on the maintenance of health and the prevention of disorders (Table 5). Settineri and Palmer [105,106] Settineri patented supplements which can be used to treat nutritional deficiencies, chronic illnesses, and syndromes, as well as to maintain lipid balance for normal mitochondrial function, among others, during increased sports performance. Robertson [107] patented a dry dietary supplement beneficial for enhancement of the human immune system. Purpura et al. [108] patented a physiologically active composition which can be used for the prevention or successful treatment of inflammatory and degenerative diseases, in particular those with a chronic course (e.g., arthritis, and arthroses). In turn, other authors [109,110,111] have developed recipes for products with a remarkable healing effect on various bone-and-joint diseases, ensuring the best recovery after exercise or a training program. Naidu et al. [112] patented a recipe for a composition which includes coenzyme Q10, lactoferrin and angiogenin suitable for multi-functional health applications. Aguilera et al. [113] patented a sport beverage containing anthocyanins which exhibits significant biological activities, including antioxidant properties, neuroprotective, anticarcinogenic, and antidiabetic functions, even related to visual acuity and dermal health. In turn, Anderson et al. [114] patented a recipe for nutrient-dense meat structured protein products, providing complete sources of protein and essential nutrients and enhancing mental performance.

Other researchers have patented products which can be applied to controlling body weight (Table 6). Chang [115] patented a functional food suitable for sportspeople based on the α-amylase inhibitor from white kidney beans. McCleary et al. [116] patented a weight loss supplement which can be used in combination with food, as a condiment, salad dressing, as well as in beverages such as sport drinks. Additionally, Udell and Israel [117] developed a recipe for a composition which can be incorporated into sport drinks and bars. In turn, Lescuyer [118] patented a slimming composition administrable orally and a dietary supplement incorporating the aforementioned composition. Other authors have patented a solid drink [119] and tablet [120] suitable for slimming and losing weight, while Badalov [121] patented a recipe for sweeteners applicable in foods and drinks.

### 3.3. An Overview of Original Scientific Articles

The majority of authors focused on the investigation of the consumption frequency of meals containing kidney beans based on the diagnostic survey method with the use of a questionnaire in female and male sportspeople practicing varied sport disciplines (Table 7). The substantial consumption of meals comprising kidney beans was reported by sportspeople from Brazil [122,123,124]; however, their acceptance by para-athletes was rather low [125]. The substantial consumption of kidney beans was recorded in sportspeople from Ireland [126], as well as Kenya [127,128,129] and the Republic of Congo [130]. Additionally, in India [131] and Iran [132] the intake of products based on kidney beans is considerable. In turn, the use of kidney beans by vegan runners from Germany, Austria, Switzerland is substantial [133], wherein female athletes exhibit a greater intake of beans than male athletes [134]. According to Vinci [135], the incorporation of beans into the diet of athletes leads to an increase in protein intake. On the other hand, investigations by several authors [136,137,138,139] showed low consumption of kidney bean by sportspeople from Australia and the UK.

Moreover, the effects of the intake of products containing kidney bean constituents were investigated by Wei [140], who showed that consumption of high-protein, low-calorie food does not influence the body height and weight of teenage male and female gymnasts.

## 4. Discussion

The increase in the number of patents in consecutive years up to year 2017 noticed in the presented study followed by an insignificant decrease in subsequent years corresponds to other findings. A similar tendency was observed previously in the case of patents containing constituents from the soybean *Glycine max* (L.) Merr. [17] and maize *Zea mays* L. [141]. Such a phenomenon may be linked to growing interest in the use of plant constituents (particularly proteins) in sport nutrition e.g., [142,143] and the literature cited here. The greatest number of patent-holders representing the United States of America and China might be linked to the considerable cultivation of *Phaseolus vulgaris* in the aforementioned countries, reflected in the increase of yearly yield documented in China since the 1960s [144] and in the United States between the years 1909 and 2012 [145]. Moreover, an increase in production of dry beans (pinto bean, navy bean, kidney bean and others) in the United States in the years 1990–2020 was reported by Siddiq et al. [146]. Such findings are consistent with studies by Uebersax et al. [147], who pointed out that, regionally, Asia leads in common bean production, followed by North, Central, and South America and Africa, while Europe and Oceania contribute only slightly to total production. The investigations conducted showed that that the majority of patents were created by research teams consisting of at least four scientists. Such a phenomenon is consistent with the worldwide tendency of the transformation of scientific research patterns in the natural sciences from individual research to teamwork [148].

The variety of patents seems to confirm the wide range of applications of common bean constituents. A substantial number of inventions concerned food ingredients. The numerous patents applying the amino acids, polypeptides and proteins derived from *Phaseolus vulgaris* in sport nutrition correspond with findings of many authors pointing out the substantial antioxidant activity of black bean protein hydrolysates [149,150] and their considerable use in the production of beverages such as sport drinks [18]. Other patents concern the use of saccharides from kidney beans in sweeteners or the use of flavonoids (such as isoflavones and anthocyanidins) occurring inter alia in fruits and seeds. At the same time, Kan et al. [151] noticed that non-white bean seed coats which contained more anthocyanidins showed much higher antioxidant activities than white ones. Considering the widely documented toxicity of some constituents of *Phaseolus vulgaris* see [37], a patent obtained by Hossen et al. [63], which presents methods to reduce saponin compound content, is very valuable. Moreover, it corresponds to other investigations addressing the efficacy of various methods of processing raw materials of leguminous plants, inter alia *Phaseolus vulgaris*. Khrisanapant et al. [152] investigated the effects of hydrothermal processing of varying durations on the texture, starch content, and protein digestibility of pulses, inter alia *Phaseolus vulgaris.* Moreover, Samtiya et al. [14] and Sharma [153] reviewed several processing techniques and methods such as fermentation, germination, debranning, autoclaving, soaking etc., which might be used to reduce the antinutrient contents in pulses. At the same time, it is worth mentioning that cooked seeds of the common bean may not be completely digested and absorbed in the bowel. The fermentation caused by bacterial activity contributes to the formation of gas and flatulence responsible for the gastrointestinal symptoms frequently reported by athletes [15].

The considerable number of patents concerning sport nutrition noticed in the review conducted here seem to confirm the findings of Arbach et al. [18] showing the frequent use of *Phaseolus vulgaris* in nutritional products. A similar phenomenon was also observed by Kostrakiewicz-Gierałt [154], who studied plant-based patents dedicated to vegan and vegetarian sportspeople. Moreover, numerous authors have patented compositions comprising *Phaseolus vulgaris* constituents suitable for relieving fatigue, enhancing endurance, as well as increasing muscle mass and strength. An improvement in athletic performance and/or adaptation to training was demonstrated as a result of consumption of supplements or meals based on other leguminous plants, among others soy, e.g., [155,156,157] and pea e.g., [158]. On the other hand, Mizelman et al. [159] (2020) stated that a pulse-based diet does not affect performance among soccer players. Patents containing constituents from *Phaseolus vulgaris* focusing on the maintenance of athletes’ health and prevention of disorders correspond to findings regarding the beneficial effects of the administration of constituents derived from other leguminous species on chronic pain, discomfort due to chondral injuries and bone resorption [160,161]. Moreover, patents maintaining the immune system of sportspeople and containing constituents from *Phaseolus vulgaris* seem to confirm the anti-inflammatory activity of legumes widely reported by Zhu et al. [162]. The recipes for compositions containing *Phaseolus vulgaris* constituents suitable for controlling body weight correspond to the findings of other authors e.g., [163,164], who demonstrated that plant-based diets comprising, among others, beans, chickpeas and soy contribute to significant weight loss in athletes. On the other hand, the investigations of Wei [140] documented a lack of change of body weight among teenager gymnasts as an effect of the consumption of high-protein, low-calorie food containing inter alia, kidney beans. Summarizing, it should be pointed out that the present literature review showed, on the one hand, a large number of patents dedicated for sportspeople but on the other hand, an insufficient number of studies focusing on the effects of the intake of products containing the kidney bean on the mental and physical health of sportspeople.

Generally, the consumption of the common bean by athletes partly reflects the main trends of the worldwide consumption of *Phaseolus vulgaris*. The substantial consumption of the common bean by athletes from India may be surprising considering the fact that, although India is a leading producer of dry beans [146], the common bean does not represent the most popular legume crop in traditional cuisine [165,166]. The acceptance of the common bean by athletes from the United States of America seems to correspond with the culinary traditions of ethnic groups [167] and the recommendations of the US Department of Agriculture [168] but is not consistent with low consumption by the US population [169]. In turn, the low rating of the common bean by athletes from Australia seems not to support the tendency of substantial consumption of *Phaseolus vulgaris* by Australian people [170]. Additionally, the sporadic intake of bean products declared by athletes from the UK does not correspond to overall consumption in the aforementioned country [171]. On the other hand, the considerable intake by athletes from Ireland, Switzerland, Austria, and Germany (particularly those following a vegan diet) might indicate an emerging tendency of more frequent consumption of plant proteins in western European food cultures, e.g., [172,173]. Moreover, the substantial consumption of beans by Brazilian athletes is consistent with the fact that *Phaseolus vulgaris* is considered to be a main pulse crop in Brazil, with old traditions of consumption and a variety of cultivars important in the local and international market [174]. Furthermore, the considerable consumption of the common bean by athletes from Kenya and The Republic of the Congo is consistent with fact that the common bean is recognised as a crop that can ensure food security in many countries of Sub-Saharan Africa [175,176]. Likewise, numerous authors have stated that the common bean is an important source of protein for both poor and wealthy households in Kenya [177] and The Republic of the Congo [178], as well as Benin [179], Burundi [180], Tanzania [181] and others. What is more, the considerable consumption of meals containing the common bean by athletes from Iran is consistent with substantial consumption of dry beans by the Iranian population, documented by Siddiq et al. [146]. The present review of literature revealed that despite the growing scientific interest in the assessment of the frequency of consumption and acceptance of meals containing the common bean, a gap in knowledge still exists. Therefore, the continuation of such investigations covering in particular Central and Eastern European, as well as Asian countries seems to be strongly needed. Cross-sectional studies among sportspeople of different nationalities representing diverse sport disciplines seems to be desirable.

To sum up, based on the performed investigations, it can be stated that food products containing *Phaseolus vulgaris* can be recommended for sportspeople and sports practitioners. The *Phaseolus vulgaris* constituents are effective for enhancing athletic endurance and recovery. The reduction or elimination of antinutritional constituents and improvement of protein digestibility during processing contribute to the high potential of the kidney bean to be used as a raw material for the manufacturing of food products (dedicated especially for sportspeople who do not suffer from intestinal problems). At the same time, it should be added that the future directions of research should concentrate on: (i) the effects of food products containing the common bean on the health of sportspeople, (ii) extensive research on the frequency of intake of meals containing the common bean, especially in the countries of Central and Eastern Europe and Asia and (iii) a comparison of the use of selected constituents derived from *Phaseolus vulgaris* and other species belonging to the Fabaceae family in the chosen kinds of food products dedicated for sportspeople, such as drinks, bars, cakes, and others.

## 5. Conclusions

The noticed increase in the number of patents up to the year 2017 related to products suitable for sportspeople containing *Phaseolus vulgaris,* followed by a slight decrease in subsequent years, may be linked to growing interest in the use of plant constituents in sport nutrition. The majority of patents developed in large research teams by scientists affiliated mainly with the United States of America and China correspond to the considerable cultivation of *Phaseolus vulgaris* in the aforementioned countries. Many patents involve the production of food ingredients and methods of processing raw leguminous materials, which is particularly important in the context of antinutrient occurrence. However, the majority of patents concern nutritional products in small forms (e.g., bars, biscuits, chips), sweet or savoury fillings, and instant powders, as well as wholesome meals. Other patents present recipes for nutritive compositions: (i) for relieving fatigue, enhancing endurance, and increasing muscle mass and strength, (ii) for maintaining physical and mental health, and (iii) for controlling body weight. The large number of patents indicates the beneficial role of constituents of the common bean in the health and recovery of sportspeople. Generally, the consumption of the common bean by athletes partly reflects the main trends of the worldwide consumption of *Phaseolus vulgaris*. In particular, the substantial consumption of the common bean by athletes from India and the USA and its sporadic use by sportspeople from Australia and the UK may be surprising. On the other hand, the considerable consumption of the common bean by athletes from Brazil, Iran, and African countries is consistent with long culinary traditions and food habits, while the substantial consumption of the common bean by athletes from Ireland, Switzerland, Austria, and Germany may indicate an emerging tendency towards plant protein consumption in western European food cultures. To summarize, the performed investigations demonstrate the substantial use of *Phaseolus vulgaris* in sport nutrition and the growing acceptance of this trend. The future directions of research should concentrate on (i) the effects of food products containing the common bean on the health of sportspeople, (ii) the frequency of intake of meals containing the common bean by sport practitioners, and (iii) a comparison of the use of selected constituents derived from *Phaseolus vulgaris* and other species belonging to the Fabaceae family in the chosen kinds of food products dedicated to sportspeople, such as drinks, bars, cakes, and others.

## Figures and Tables

**Table 1:** The mean (±SD) number of patents containing constituents deriving from *Phaseolus vulgaris* L. suitable for sportspeople per year issued in consecutive periods. The different letters in superscript mean statistically significant differences.

Period	Mean (±SD)	The H Kruskal-Wallis Test, *p*-Value
1995–2001	0.29 (±0.49) ^a^	H = 14.69, *p* < 0.01
2002–2008	1.86 (±1.35) ^ab^	
2009–2015	4.86 (±2.61) ^b^	
2016–2023	4.00 (±3.11) ^b^	

**Table 2:** A review of patents and inventions of food ingredients containing constituents derived from *Phaseolus vulgaris* L. suitable for sportspeople.

Inventor(s)	First Author Affiliation	Year	Patent/Article Title	Reference
Kramer, R., Nikolaidis, A.	US	2011	Amino acid compositions	[41]
Silver, N. et al.	US	2014	Nutritive polypeptides, formulations and methods for treating disease and improving muscle health and maintenance	[42]
Schweizer, M., Segall, K.I.	CA	2011	Production of soluble protein solutions from pulses	[43]
Berry, D.A. et al.	US	2013	Charged nutritive proteins and methods	[44]
Berry, D.A et al.	US	2013	Nutrient Fragments, Proteins, and Methods	[45]
Silver, N.W. et al.	US	2013	Nutritive Fragments, Proteins and Methods	[46]
von Maltzahn, G. et al.	US	2013	Nutritive Proteins and Methods	[47]
Bourcier, B et al.	FR	2013	Complex of at least one vegetable protein and at least one milk protein	[48]
Keizer, L. et al.	US	2017	The component and its preparation process of product analogue or this kind of analogue	[49]
Zhang, H. et al.	US	2020	Methods for the preparation of a plant protein composition	[50]
Boursier, B., Passe, D.	FR	2010	Granulated powder containing vegetable proteins and maltodextrins, method for producing same, and uses thereof	[51]
van der Hijden, H.T. et al.	NL	2007	Food Product and Process for Preparing It	[52]
Slimak, K.M.	US	1998	Products from sweet potatoes, cassava, edible aroids, amaranth, yams, lotus, potatoes and other roots, seeds and fruit	[53]
Kannar, D. et al.	AU	2009	Process for the manufacture of sugar and other food products	[54]
Prakash, I. et al.	US	2014	Steviol glycosides, their compositions and their purification	[55]
Kumar, M., Nijmeijer, M.	NL	2015	Steviol glycosides	[56]
Markosyan, A. et al.	AM	2020	Methods of preparing steviol glycosides and uses of the same	[57]
Luo, R.Z. et al.	US	2016	Compounds, compositions, and methods for modulating sweet taste	[58]
Shi, J. et al.	CN	2020	Sweetener and flavour compositions, methods of making and methods of use thereof	[59]
Prakash, I., Dubois, G.E.	US	2007	High-potency sweetener composition with c-reactive protein reducing substance and compositions sweetened therewith	[60]
Prakash, I. et al.	US	2015	Compositions and methods using rebaudioside X to provide sweetness enhancement	[61]
Eidenberger, T	AT	2008	Stabilized anthocyanin compositions	[62]

**Table 3:** A review of patents and inventions enhancing nutrition containing constituents derived from *Phaseolus vulgaris* L. suitable for sportspeople.

Inventor(s)	First Author Affiliation	Year	Patent/Article Title	Reference
Abdel-Salam F.F. et al.	EG	2022	Formulation and evaluation of high energy-protein bars as a nutritional supplement for sports athletics	[64]
Sparvoli, F. et al.	IT	2017	Highly nutritious biscuits with common bean flours	[65]
Li, M. et al.	CN	2019	High protein sport nutrition meal substitute biscuit and preparation method thereof	[66]
Chang, A.	US	2015	Ready to eat cold vegetable cakes with ingredients	[67]
Kovalchuk T.G.	RU	2020	Whole-grain protein chips and their production method	[68]
Medina, S., Segal K.I.	CA	2014	Frozen dessert mixes using pulse protein products	[69]
Obrecht, J., Laperche, S.R.	CH	2010	Nutritious beverage and its production method	[70]
Greenberg, N.A. et al.	US	2012	Nutritional compositions comprising alpha-hydroxyisocaproic acid	[71]
Smith, R.A.	US	2012	Organic vegan protein shaker	[72]
Chang, A.	US	2014	Beverage system, including bubble beverage, instant beverage, beverage with dissolved gas, and beverage with ingredient	[73]
Prakash, I. et al.	US	2014	Beverages containing rare segars	[74]
Biyun, Z.	CN	2015	Instant drink comprising bubbles and dissolved gas and drink system	[75]
Methner, F.-J. et al.	DE	2016	Sport beverages and methods for their production	[76]
Yinglei, Z. et al.	CN	2016	Vinegar beverage using black rice and black beans as basic raw materials and processing technology thereof	[77]
Kizer, L. et al.	US	2017	Product analogues or components of such analogues and processes for making same	[78]
He, W.	CN	2020	Natural nutritive making method of navel orange wine	[79]
Smith, W.C. et al.	US	2010	Functional food paste	[80]
Hongtao, L.	CN	2003	Multiple element nutritive powder	[81]
Li, X.	CN	2017	Peptide-containing meal powder and processing method thereof	[82]
Lei, S. et al.	CN	2022	Instant cereal product and preparation method thereof	[83]
Tao, T., Ting, L.	CN	2017	Quinoa nutritive meal replacement powder	[84]
Boursier, B. et al.	FR	2014	Assembly of at least one vegetable protein and at least one dairy protein	[85]
Bolster, D. et al.	US	2012	Nutritional compositions including branched chain fatty acids and methods of using same	[86]
Bolster, D. et al.	US	2022	Plant-based protein mixtures and nutritional compositions	[87]
Hangjian, G.	CN	2006	Nutrition food	[88]
Xu, H.	CN	2014	Nutritional food useful for athletes comprises corn flour, Astragalus root powder, colla corii asini, small red bean powder, flour, propolis, honey, aspartame and vinegar	[89]
Savant, V.D. et al.	US	2013	Puree compositions having specific carbohydrate ratios and methods for using same	[90]
Nardelli Junior G.P.	BR	2017	Nutritional food supplement based on wet cassava starch modified by hydration or rehydration, also known as tapioca, used as a carbohydrate source and the basis for formulations of dieting or nutritional food supplements, and also as partial substitute for meals of athletes or persons practicing physical activities, its compositions and production process	[91]

**Table 4:** A review of patents relieving fatigue, enhancing endurance, as well as increasing muscle mass and strength, containing constituents derived from *Phaseolus vulgaris* L. suitable for sportspeople.

Inventor(s)	First Author Affiliation	Year	Patent/Article Title	Reference
Zhan, Y. et al.	CN	2018	Anti-fatigue sport type salt soda	[92]
Tao, S.	CN	2019	Functional bean paste containing polyphenol and preparation method thereof	[93]
Hageman, R.J.J. et al.	NL	2012	Combination of components for the prevention and treatment of frailty	[94]
De Wilde, M.C. et al.	NL	2012	Non-medical increase or maintenance of body weight of a mammalian	[95]
Liao, G	CN	1995	Health-care nutritious drink for fatigue resistance and strengthen endurance for sport	[96]
Choi, Ch., W. et al.	KR	2004	Functional food composition which comprises cereals, fruits and vegetables, seaweeds and mushrooms useful and enhances physical strength and exercise performance of athlete and boosting fatigue recovery	[97]
Fan, Y., Feng, Q.	CN	2017	Sweet sour bean drink and production method thereof	[98]
Scheiman, J. et al.	US	2020	Compositions and methods for enhancing exercise endurance	[99]
Xu, X. et al.	CN	2022	Use of menaquinone-7 in preparing products for improving athletic ability in patients with diabetes, sports persons and muscle attenuation people, where product is pharmaceutical, food or health product, and food product is dairy product, an edible oil, bean product	[100]
Xu, B.	CN	2021	Solid beverage beneficial to body shaping and preparation method thereof	[101]
Bailey, D.M. et al.	CH	2016	Methods for enhancing muscle protein synthesis following concurrent training	[102]
Veen, M., Budemann, A.	DE	2013	Food composition containing amino acids and cocoa	[103]
Longo, V. et al.	US	2023	A diet composition for enhancing lean body mass and muscle mass	[104]

**Table 5:** A review of patents maintaining health and preventing disorders containing constituents derived from *Phaseolus vulgaris* L. suitable for sportspeople.

Inventor(s)	First Author Affiliation	Year	Patent/Article Title	Reference
Settineri, R.A., Palmer, J.F.	US	2012	Lipid supplements for maintaining health and the treatment of acute and chronic disorders	[105]
Settineri, R.	US	2015	Flavoured chewable lipid supplements for maintaining health and the treatment of acute and chronic disorders	[106]
Robertson, M.	US	2006	Universal protein formulation meeting multiple dietary needs for optimal health and enhancing the human immune system	[107]
Purpura, M. et al.	DE	2006	Physiologically-Active Composition Based on Collagen	[108]
Li, A., Xu, Q.	CN	2004	Joint-protecting beverages and/or foods and their preparations	[109]
Fang, K. et al.	CN	2016	Functional nutrient food for improving joints	[110]
Feng, W. et al.	US	2018	Composition and production and preparation method thereof for sports achievement	[111]
Naidu, A.S. et al.	US	2007	Coenzyme Q10, lactoferrin and angiogenin compositions and uses thereof	[112]
Aguilera Y. et al.	ES	2016	Black bean coats: New source of anthocyanins stabilized by β-cyclodextrin co-pigmentation in a sport beverage	[113]
Anderson, D. et al.	US	2015	Nutrient-dense meat structured protein products	[114]

**Table 6:** A review of patents controlling body weight containing constituents derived from *Phaseolus vulgaris* L. suitable for sportspeople.

Inventor(s)	First Author Affiliation	Year	Patent/Article Title	Reference
Chang Ch.	CN	2017	Development of nanometre lipid functional food and its influence on sports and health industry	[115]
McCleary, E. et al.	US	2005	Foods, beverages, condiments, spices and salad dressings with specialized supplements	[116]
Udell, R., Israel, K.	US	2006	Nutritional supplement for body fat reduction	[117]
Lescuyer, J.F.	FR	2010	Dieting composition, useful for reducing weight and as dietary supplement, preferably for sportsman comprises epigallocatechin gallate, capsaicin, caffeine, tyrosine and extract of white bean (*Phaseolus vulgaris*)	[118]
Jin, Y.	CN	2017	Slimming and weight losing solid drink	[119]
Song, L. et al.	CN	2022	Sports nutrition tablet for losing weight and preparation method thereof	[120]
Badalov, C.	CA	2008	Super sweet sugar crystals and syrups for health and method	[121]

**Table 7:** A review of original articles devoted to the frequency and time of eating of *Phaseolus vulgaris* L. by sportspeople in alphabetical order. Abbreviations: Gender: F-female, M-male, Country: Australia (AU), Austria (AT), Brazil (BR), The Republic of the Congo (CG), Germany (DE), India (IN), Iran (IR), Ireland (IE), Kenya (KE), Switzerland (CH), United Kingdom (UK), United States of America (US).

References	Physical Activity	Age (Years)	Gender	Country	Results
Noll et al. [122]	Soccer, handball, volleyball, basketball	14–20	M	BR	82% of athletes eat dishes containing beans 5–7 days per week. The lowest bean consumption was declared by soccer players
Guerra et al. [123]	Handball	12–14	F	BR	78.6% of respondents consume bean products
Santos et al. [124]	Soccer	20.8 ± 4.5	F	BR	40% of athletes consume dishes containing beans approximately 7 days per week
Sasaki et al. [125]	Para-athlete team disciplines (basketball, cerebral palsy football, badminton, rugby, wheelchair tennis, sitting volleyball)	18–60	F, M	BR	The consumption of beans is rather low. The consumption of beans is greater in para-athletes in team sports than among individual sports para-athletes
Walsh et al. [126]	Rugby	15–18	M	IE	80.8% players consume high-protein foods (containing beans) before exercise
Christensen et al. [127]	Middle- and long-distance running	15–20	M	KE	81% use kidney beans as a staple food
Onywera et al. [128]	Middle- and long-distance running	19–24	.	KE	Beans are staple food
Waititu et al. [129]	Middle- and long-distance running	18–26	F, M	KE	Substantial consumption of dishes containing kidney beans
Mabossy-Mobouna et al. [130]	Various sport disciplines (athletics, soccer, handball, judo, karate and others)	18–48	F, M	CG	Beans are the most consumed legume
Nazni, Vimala [131]	Running, volleyball, weightlifting	19–24	M	IN	26% of sportspeople confirm consumption of products containing beans one time per day
Noormohammadpour et al. [132]	Soccer	10–34	M	IR	35.6% respondents consume bean products 2–3 times per week
Wirnitzer et al. [133]	Long-distance running	≥18	F, M	AT, DE, CH	Substantial use of beans by vegan runners
Motevalli et al. [134]	Long-distance running	≥18	F, M	AT, DE, CH	Females consume more beans than males
Vinci [135]	Golf	.	F	US	Incorporation of beans into the diet to increase protein intake
Keith et al. [136]	Cycling	.	F	.	Low consumption of several products including beans
Alaunyte et al. [137]	Rugby	18–36	M	UK	The majority of players declare rare consumption of bean products
Tam et al. [138]	Anaerobic-power sports	21.5 ± 4.5	F	AU	Low rating of nutritional value of kidney beans by 76% of athletes.
Feng, Yuan [139]	Trampoline athletes	10–17	F, M	.	Low consumption of several products including beans

## Data Availability

Not applicable.
